# The relationship between the oblique sagittal temporomandibular joint disc position and the volume surface area of the condyle in young TMD adults

**DOI:** 10.3389/fbioe.2023.1321241

**Published:** 2023-12-21

**Authors:** Yudong Gao, Dan Luo, Mujie Yuan, Yanhao Yang, Zexian Xu, Jianjun Yang

**Affiliations:** ^1^ The Affiliated Hospital of Qingdao University, Qingdao, China; ^2^ School of Stomatology, Qingdao University, Qingdao, China; ^3^ Dental Digital Medicine and 3D Printing Engineering Laboratory of Qingdao, Qingdao, China

**Keywords:** temporomandibular joint disc, condyle apex method, the volume of the condyle, the surface area of the condyle, MRI, CBCT

## Abstract

The present study aims to compare the volume surface area of the condyle, the horizontal condylar axial angle and the disc-condyle angle between temporomandibular disorder (TMD) and asymptomatic volunteers, explore and analyze the relationship between the temporomandibular joint (TMJ) disc position in oblique sagittal plane and the volume surface area of the condyle in young adults with TMD symptoms. 84 young adult volunteers were received TMJ examination by Magnetic Resonance Imaging (MRI) and Cone Beam Computed Tomography (CBCT). TMD and asymptomatic volunteers were 42 each. MRI was used to assess the position of TMJ disc in the oblique sagittal plane with the condyle apex method. CBCT data were used for three-dimensional (3D) reconstruction of condyle and the measurements of the horizontal condylar axial angle and the volume surface area of the condyle. The condylar volume surface area of the TMD group was smaller than that of the asymptomatic group (*p* < 0.05), the disc condyle angle was larger than that of the asymptomatic group (*p* < 0.05), and no significant difference was found in the horizontal condylar axial angle (*p* > 0.05). In terms of correlation, the volume surface area of the condyle were negatively correlated with the position of the articular disc in TMD patients (*p* < 0.05). This significant negative correlation suggests that the possibility of disc displacement can be considered when poor condylar morphology is found.

## 1 Introduction

Mandibular condylar growth center can respond adaptively to stimulation of the surroundings through bone rebuilding. This adaptive response plays an important role in the growth and development period of individual maxillofacial growth stability (Krisjane et al., 2007), while in adulthood, it may cause changes in condylar bone such as flattening, sclerosis, osteophyte formation and erosion, resorption of the condylar head (Seo et al., 2022), thus changing the size and morphology of the condyle. Some researchers believe that anterior disc displacement alters the microenvironment of the condyle, which may irritate to impair condylar growth and remodeling (Xie et al., 2016; Yasa and akgül, 2018; Liu et al., 2023). In clinical practice, we have also found that condyle morphology changes in some TMD patients, such as the condyle of patients with joint click symptoms may show flattening in oblique sagittal CBCT, the bilateral condyle of patients with mandibular dyskinesia may show inconsistent size, and the condyle osteophyte formation and erosion of patients with pain symptoms. On subsequent MRI examination, we also found that these patients were accompanied by various degrees of changes in the position of the articular disc.

However, most previous studies have used linear or angular indices to assess the morphology of the condyle (Rabelo et al., 2017; de Pontes et al., 2019; Yildizer and Odabaşı, 2023), necessitating an examination of the disc-condyle relationship from the perspective of volume surface area of the condyle. Furthermore, our research group previously proposed that the condyle apex method was more accurate than the condyle center method in measuring the disc-condyle angle, suggesting that the position of the articular disc in asymptomatic young adults should be in the anterosuperior region of the condyle (Luo et al., 2022). This method was continued in this study to assess the angle of the articular disc in an oblique sagittal direction in young volunteers. In addition, as the mandibular condyle is divided into left and right sides, the horizontal condylar axial angle on both sides was also measured.

The aim of this study was to compare the volume surface area of the condyle, the horizontal condylar axial angle and the disc-condyle angle between TMD and asymptomatic volunteers, and to analyze the relationship between the position of articular disc in oblique sagittal plane and the volume surface area of the condyle in young TMD adults.

## 2 Materials and methods

### 2.1 Sample selection

Eighty-four students were selected from a university in Qingdao. The inclusion criteria of TMD volunteers were as follows: 1) volunteers were aged over 18; 2) volunteers had TMD symptoms such as joint clicking when opening or closing mouth through the examination of TMD specialists; 3) volunteers underwent MRI and CBCT examinations simultaneously. Exclusion criteria were 1) systemic diseases affecting bones; 2) TMJ surgery history or Congenital odontomaxillofacial deformity; 3) Malocclusion.

The inclusion and exclusion of asymptomatic volunteers was referred to the criteria of Luo (Luo et al., 2022). Inclusion criteria were 1) age >18 years; 2) no signs or symptoms of a TMD, as confirmed by an experienced clinical specialist; 3) the acquisition of CBCT and MRI in the oblique sagittal of the bilateral TMJ in closed-mouth position; 4) Consistent with individual normal occlusion. Exclusion criteria were 1) rheumatoid arthritis in childhood or systemic inflammatory arthritis; 2) any contraindication to CBCT and MRI due to a general condition such a claustrophobia or metal implants.

The study was approved by the Ethics Committee of the Affiliated Hospital of Qingdao University (NO. QYFYWZLL 27452). All volunteers gave written informed consent.

### 2.2 MRI acquisition and measurement analysis

MRI was used to examine the position of TMJ disc in the oblique sagittal plane with the condylar apex method. Images acquisition were executed by the Siemens Magnetom Prisma 3.0T MRI system (Siemens, Erlangen, Germany), with a 64-channel head coil that captures the global spatial information of the TMJ muscle and soft tissue structures. The volunteers were instructed to assume a supine position, while keeping the articular disc position in the oblique sagittal plane in the closed mouth position. The TMJ OSAG-PDW-FSE sequences were obtained by three-dimensional (3D) volume scanning and parallel acquisition technology, with an excitation time of 3s, repetition time (TR) of 2070 m, echo time (TE) of 28 m, field of view (FOV) of 120 × 120 mm, matrix of 192 × 144, and plane resolution of 0.6 × 0.6. A total of 16 images were taken (left and right TMJ, eight images each). The layer thickness of each one was 2 mm and the layer spacing 10%. Two dental imaging specialists with no prior knowledge of the sample data independently measured the disc condyle angle.

**FIGURE 1:**
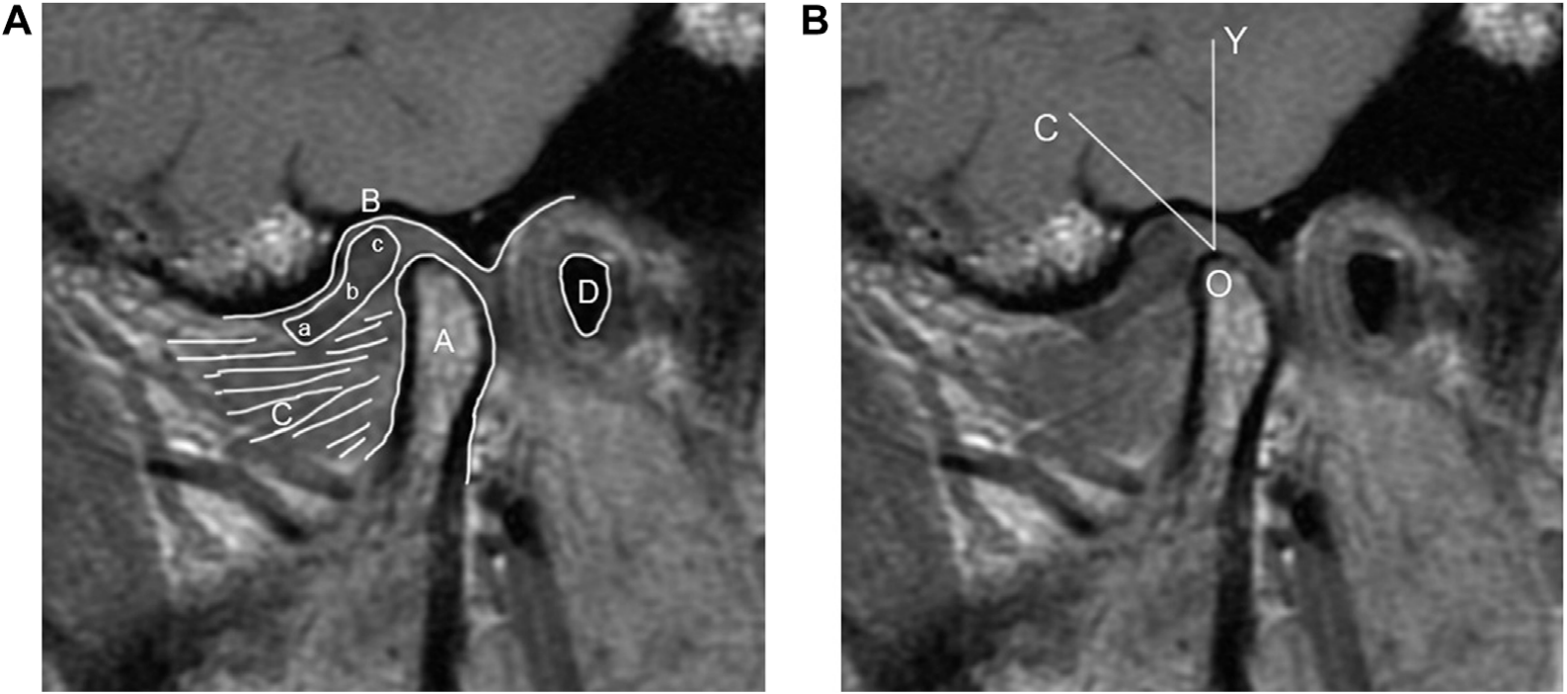
**(A)** MRI diagram of oblique sagittal joint structure. (A) Condyle; (B) glenoid fossa; (C) Lateral pterygoid muscle; (D) External auditory meatus; (a) anterior band; (b) intermediate zone; (c) posterior band. **(B)** Schematic diagram of the condyle apex method (O) the apex of the condyle (O–Y) a line perpendicular to the axial plane (O–C) a line connecting the apex of the condyle and the posterior edge of the articular disc posterior band (∠COY) disc condyle angle.

The condyle apex method, simply stated, entails first determining the apex of the condyle and drawing a line perpendicular to the axial plane (The axial plane of MRI is the plane perpendicular to the horizontal ground) through it, and then determining the posterior edge of the articular disc posterior band and connecting it to the apex of the condyle. The angle formed by the two lines can be used to determine where the articular disc is located (Figure 1).

### 2.3 CBCT acquisition and measurement analysis

CBCT was used to access the condylar bone condition, and i-CAT scanner (Imaging Sciences International 17–19, Hatfield, PA, United States) was adopted with the following parameters: 120KV, 5mA, exposure time of 26.9 s, image matrix size of 640 × 640, voxel size of 0.25 mm, and field of view (FOV) of 16.0 × 11.0 cm. All participants had their CBCT images acquired by a radiologist with the same parameter settings. They were required to sit up straight with their mandibles kept in the intercuspal position, the Frankfurt plane paralleled to the horizontal ground, and the midsagittal plane of the head perpendicular to the horizontal ground. The CBCT image data obtained were saved in a Digital Imaging and Communications in Medicine (DICOM) format.

**FIGURE 2:**
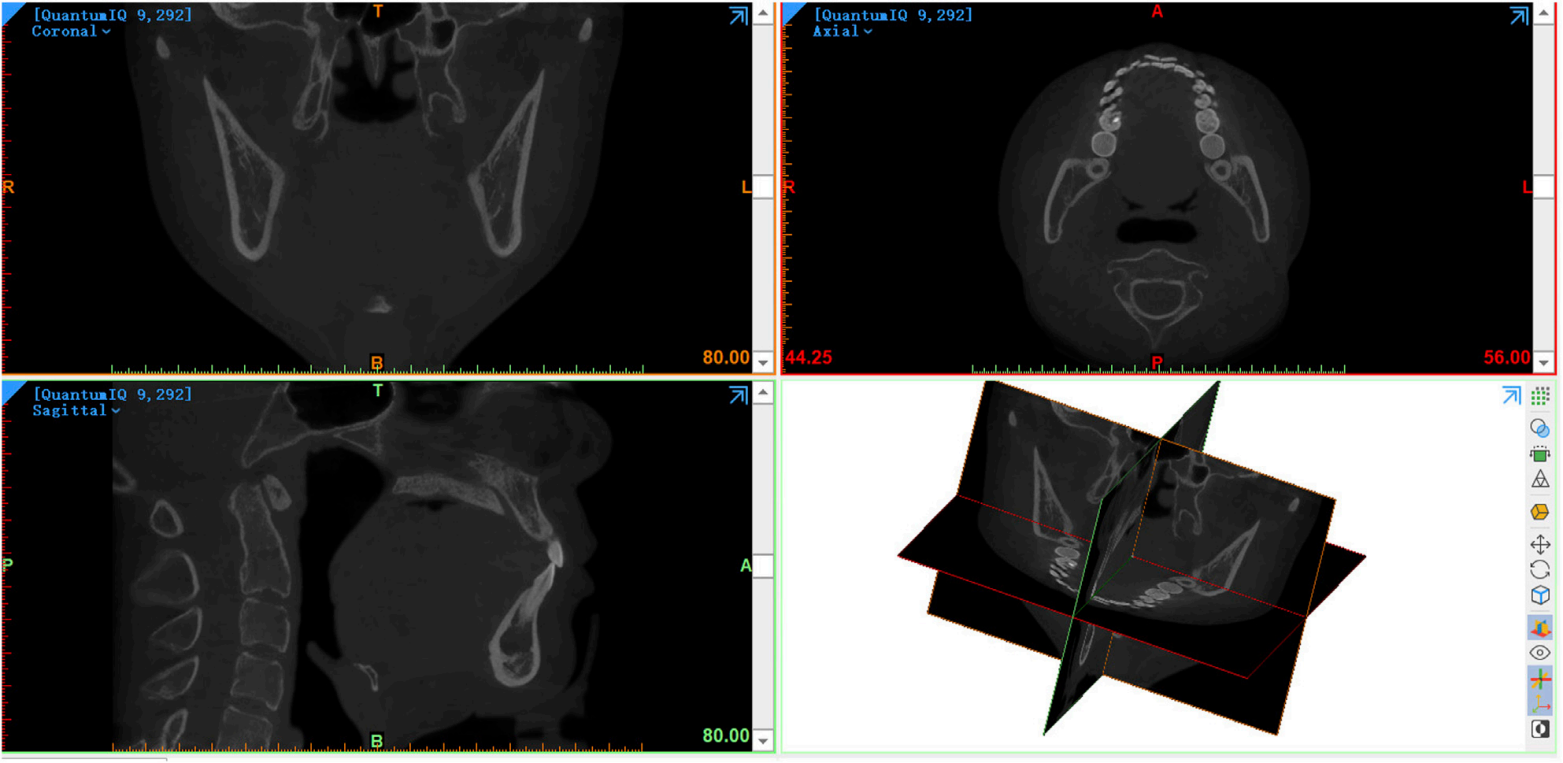
The CBCT data in DICOM format was imported into mimics21 software.

The CBCT data in DICOM format was then imported into mimics21 (Materialise, Leuven, Belgium) software for 3D reconstruction of condyles (Figure 2). After setting the direction, the shape and size of the craniofacial bone could be observed in the coronal plane, sagittal plane and axial plane. The operator selected the software’s gray value range (226–3,071) of the bone for the first mask creation. At this point, the mask of skull and mandibular condyle can been observed.

**FIGURE 3:**
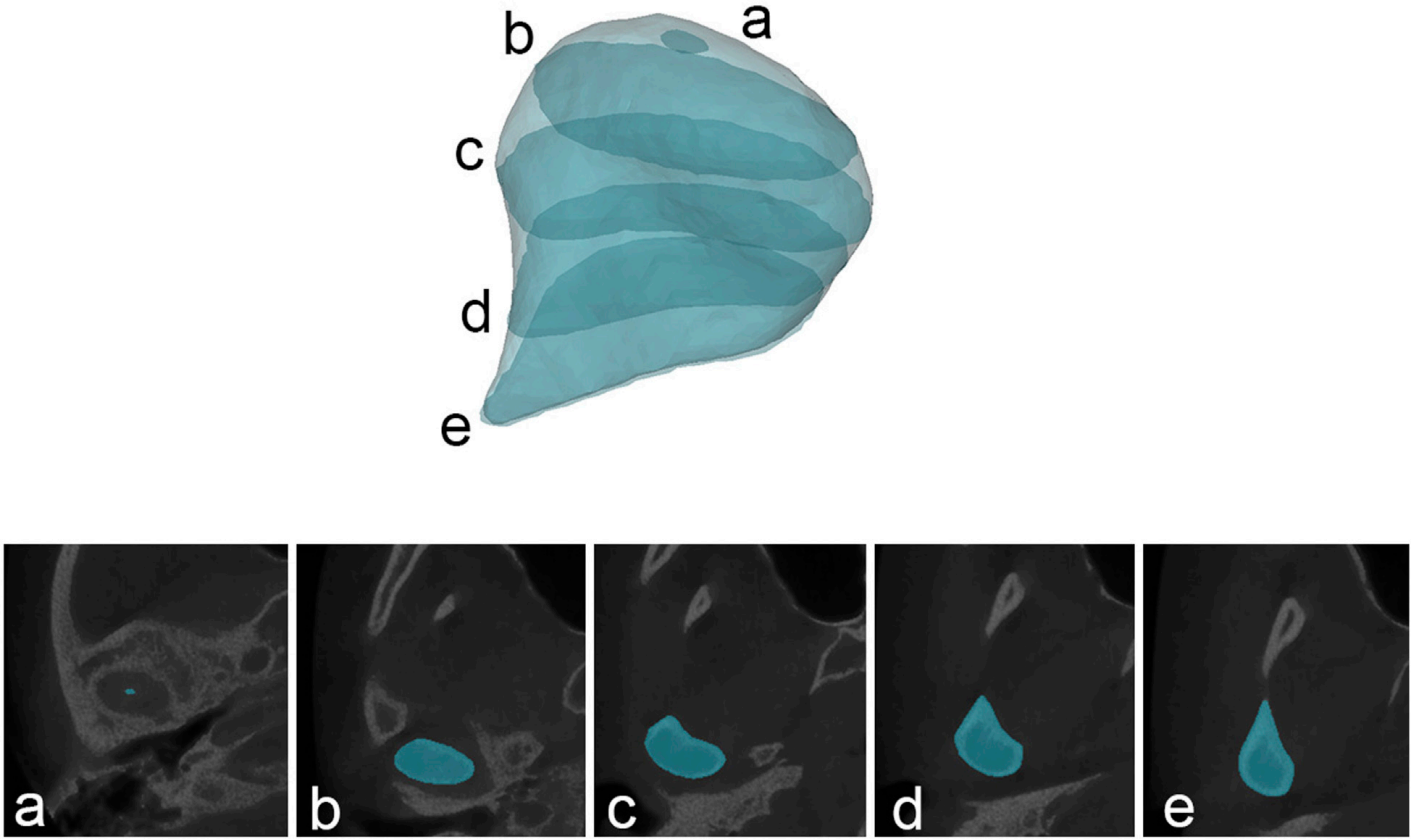
The shape of the condylar mask at different levels. **(A)** the upper condylar boundary; **(B)** long ellipse mask; **(C)** elliptic mask; **(D)** water-drop mask; **(E)** the lower boundary of the condyle.

The anatomical structure of the condyle was divided according to the method mentioned by Tecco et al. (Tecco et al., 2010). In the axial plane (The axial plane of CBCT is the plane parallel to the horizontal ground), the mask was slid sequentially from top to bottom until the first radiopaque point in the articular fossa was observed, defining this as the upper condylar boundary. When the interface between the condyle and the coracoid process was observed, the upper layer of the condyle mask of this interface was defined as the lower boundary of the condyle. The outer shape of the mask can be clearly observed between the upper and lower boundaries of the condyle, ranging from long ellipse to ellipse and then to water-drop roughly (Figure 3).

**FIGURE 4:**
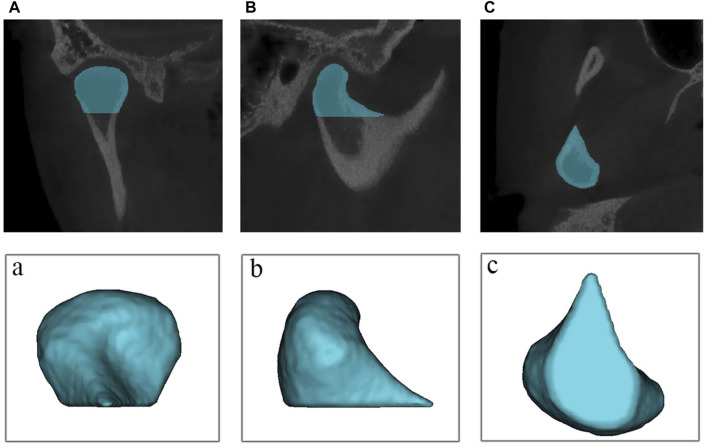
The reconstruction of 3D model of condyle**. (A)** coronal view of the mask **(B)** sagittal view of the mask **(C)** overhead view of the mask; (a) coronal view of the model; (b) sagittal view of the model; (c) sagittal view of the model.

The Region Growing tool was used to select a point from the original mask’s condylar region for the second mask creation. The mask of the condyle was thus separated from the mask of the majority of the skull. The surface quality of the final condyle mask was therefore improved by viewing the condylar mask layer by layer, filling the internal cavity, and smoothing the irregular artifacts on the outside. The final step was to calculate part from the condyle mask. Following the completion of the reconstruction of 3D model of condyle (Figure 4), the software automatically calculated the volume and surface area of the condyle.

**FIGURE 5:**
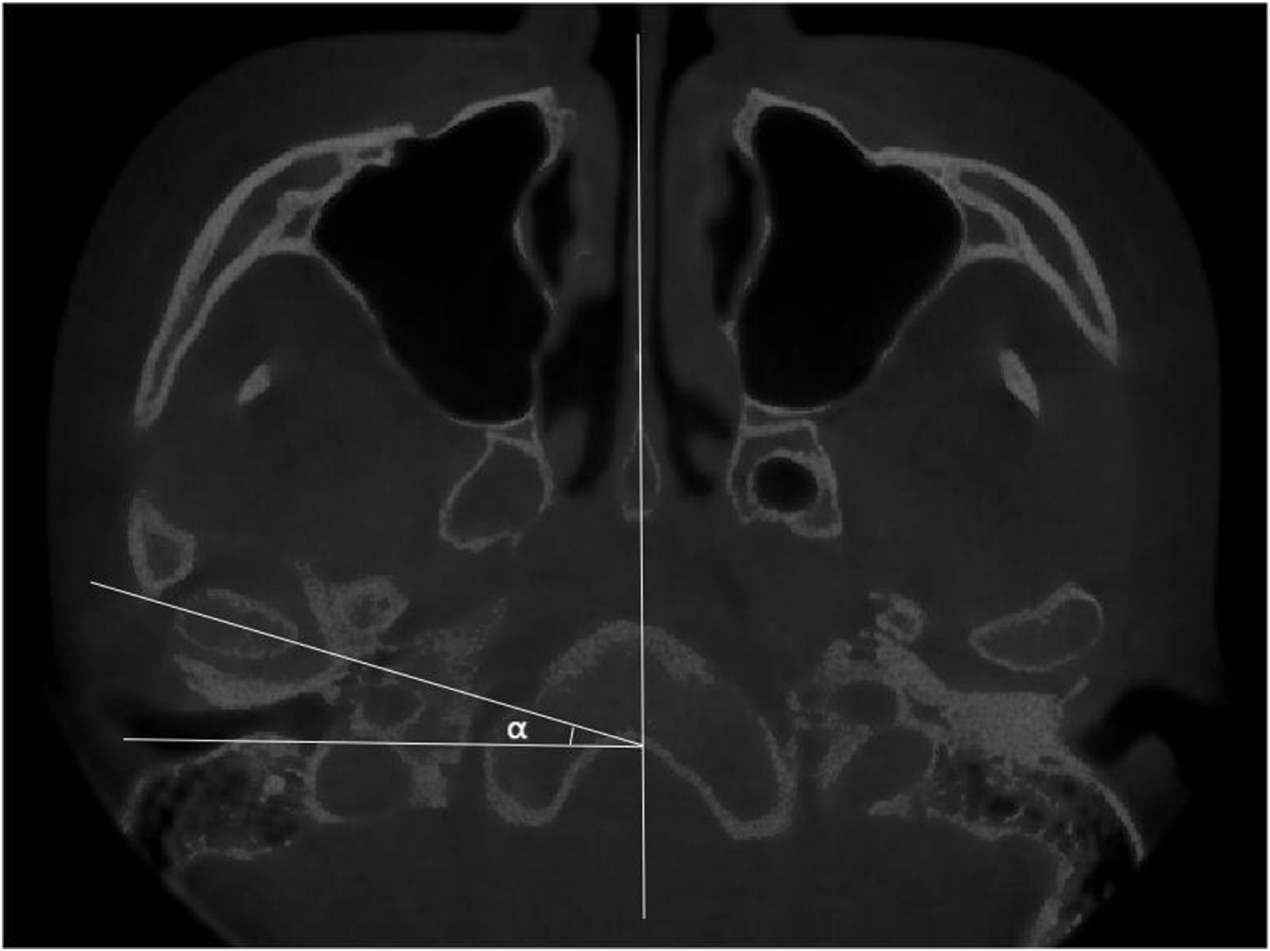
The measurements of the horizontal condylar axial angle (*α*).

On the axial plane, the layer with the largest area of the condyle was selected as the measurement plane, and a line connecting the inner and outer poles of the condyle was made. The angle between this line and the perpendicular line of the sagittal axis was measured, that is, the horizontal condylar axis angle (Figure 5).

### 2.3 Statistical analysis

SPSS 27 software (SPSS; IBM, Chicago, IL) was adopted to conduct statistical analysis, and *p* < 0.05 was considered statistically significant. Shapiro-Wilk testing confirmed normal distribution of all data. Data were expressed in terms of the maximum, minimum, mean, and standard deviation. Paired *t*-test for comparing data from different genders and sides within the same group, and two-sample t-tests was used to compare data between two groups. In terms of analyzing the correlation between the disc condyle angle and the volume surface area of the condyle, pearson test was employed, if the data conformed to the normal distribution; otherwise, the Spearman test was used. To test the magnitude of the measurement error, 20 joints were randomly selected for measurement and then measured again 4 weeks after the first measurement. The reliability of measurement had intraclass correlation coefficients higher than 0.95.

## 3 Results

A total of 168 joints were analyzed, with 84 each in asymptomatic and TMD volunteers. The age distribution of the two groups were shown in Table 1. Age difference was not statistically significant (*p* ˃ 0.05).

**TABLE 1:** The age distribution of the two groups (years old).

	Asymptomatic volunteers (n = 42)				TMD volunteers (n = 42)				*p*-value
	Min	Max	Mean	SD	Min	Max	Mean	SD	
**Age**	20	25	23.07	1.31	20	26	23.51	1.56	˃ 0.05
**Gender**									˃ 0.05
Male	20	25	23.15	1.46	20	26	23.45	1.84	
Female	21	25	23.09	1.19	21	25	23.54	1.29	

SD, standard deviation; n, number of people.

Table 2 have shown the condylar volume of asymptomatic and TMD volunteers. In the asymptomatic group, the condylar volume was significantly larger in males than that in females (*p* ˂ 0.05), however, no significant difference was found between the left and right sides (*p* ˃ 0.05). In the TMD group, the condylar volume was significantly larger in males than that in females (*p* ˂ 0.05), the left condylar volume was significantly larger than the right (*p* ˂ 0.05). Condylar volume was significantly reduced in the TMD group compared to the asymptomatic group, regardless of gender and side (*p* ˂ 0.05).

**TABLE 2:** Condylar volume of asymptomatic and TMD volunteers (mm^3^).

	Asymptomatic volunteers				TMD volunteers				*p*-value
	Min	Max	Mean	SD	Min	Max	Mean	SD	
**Total** (n = 84)	1,255.19	30.61.41	2031.09	390.62	1,056.89	2,887.34	1733.03	418.57	˂ 0.05
**Gender**									˂ 0.05
Male (n = 40)	1,546.94	2,836.89	2,199.17	311.43	1,124.76	2,887.32	1929.68	465.52	
Female (n = 44)	1,255.19	3,061.41	1878.28	395.46	1,056.89	2,120.94	1,551.26	281.97	
*p*-value	˂ 0.05				˂ 0.05				
**Side**									˂ 0.05
Left (n = 42)	1,255.19	2,836.89	2049.53	399.01	1,117.67	2,887.32	1826.28	389.43	
Right (n = 42)	1,325.81	3,061.41	2012.64	386.01	1,056.89	2,563.24	1,637.74	429.44	
*p*-value	˃ 0.05				˂ 0.05				

SD, standard deviation; n, number of joints.

Table 3 have shown the condylar surface area of asymptomatic and TMD volunteers. In the asymptomatic group, the condylar surface area was significantly larger in males than that in females (*p* ˂ 0.05), however, no significant difference was found between the left and right sides (*p* ˃ 0.05). In the TMD group, the condylar surface area was significantly larger in males than that in females (*p* ˂ 0.05), the left condylar surface area was significantly larger than the right (*p* ˂ 0.05). Condylar surface area was significantly reduced in the TMD group compared to the asymptomatic group, regardless of gender and side (*p* ˂ 0.05).

**TABLE 3:** Condylar surface area of asymptomatic and TMD volunteers (mm^2^).

	Asymptomatic volunteers				TMD volunteers				*p*-value
	Min	Max	Mean	SD	Min	Max	Mean	SD	
**Total** (n = 84)	684.05	1,484.16	979.36	179.52	592.14	1,499.35	858.59	183.51	˂ 0.05
**Gender**									˂ 0.05
Male (n = 40)	822.94	1,484.16	1,083.69	166.48	655.99	1,499.35	946.85	192.45	
Female (n = 44)	684.05	1,266.52	894.67	134.35	592.14	1,205.64	775.81	136.74	
*p*-value	˂ 0.05				˂ 0.05				
**Side**									˂ 0.05
Left (n = 42)	684.05	1,422.68	988.56	174.66	592.14	1,499.35	886.79	183.87	
Right (n = 42)	714.71	1,484.16	970.16	185.91	595.23	1,226.93	830.41	180.92	
*p*-value	˃ 0.05				˂ 0.05				

SD, standard deviation; n, number of joints.

Table 4 have shown the disc condyle angle of asymptomatic and TMD volunteers. In the asymptomatic group, there were no significant gender and side differences (*p* ˃ 0.05), while in the TMD group, the disc condyle angle in females was significantly larger than that in males (*p* ˂ 0.05), the right disc condyle angle was significantly larger than the left (*p* ˂ 0.05). Compared with the asymptomatic group, the disc condyle angle was significantly larger in the TMD group, regardless of gender and side (*p* ˂ 0.05).

**TABLE 4:** The disc condyle angle of asymptomatic and TMD volunteers (°).

	Asymptomatic volunteers				TMD volunteers				*p*-value
	Min	Max	Mean	SD	Min	Max	Mean	SD	
**Total** (n = 84)	5.2	56.7	24.27	9.29	8.4	103.6	48.86	19.82	˂ 0.05
**Gender**									˂ 0.05
Male (n = 40)	5.2	38.5	23.51	7.51	8.4	103.6	43.24	19.91	
Female (n = 44)	8.1	56.7	25.21	11.06	12	101.4	53.51	18.96	
*p*-value	˃ 0.05				˂ 0.05				
**Side**									˂ 0.05
Left (n = 42)	5.2	56.7	23.87	9.39	8.4	101.4	45.48	19.38	
Right (n = 42)	7.5	45.6	24.66	9.27	10.3	103.6	52.24	19.91	
*p*-value	˃ 0.05				˂ 0.05				

SD, standard deviation; n, number of joints.

Table 5 have shown the horizontal condylar axial angle of asymptomatic and TMD volunteers. In the asymptomatic group, there were no significant gender and side differences (*p* ˃ 0.05), while in the TMD group, the horizontal condylar axial angle in females was significantly larger than that in males (*p* ˂ 0.05), no significant difference was found between the left and right sides (*p* ˃ 0.05). Compared with the asymptomatic group, the horizontal condylar axial angle was significantly larger in the TMD group, regardless of gender and side (*p* ˂ 0.05).

**TABLE 5:** The horizontal condylar axial angle of asymptomatic and TMD volunteers (°).

	Asymptomatic volunteers				TMD volunteers				*p*-value
	Min	Max	Mean	SD	Min	Max	Mean	SD	
**Total** (n = 84)	10.1	30.2	19.15	4.48	10.5	33.2	23.01	4.78	˂ 0.05
**Gender**									˂ 0.05
Male (n = 40)	10.1	27.5	18.71	4.36	10.5	31.8	21.31	4.73	
Female (n = 44)	10.2	30.2	19.44	4.76	15.3	33.2	24.52	4.33	
*p*-value	˃ 0.05				˂ 0.05				
**Side**									˂ 0.05
Left (n = 42)	10.1	30.2	19.19	4.82	12.9	30.4	23.42	4.53	
Right (n = 42)	10.2	28.2	19.11	4.18	10.5	33.2	22.57	5.03	
*p*-value	˃ 0.05				˃ 0.05				

SD, standard deviation; n, number of joints.

**FIGURE 6:**
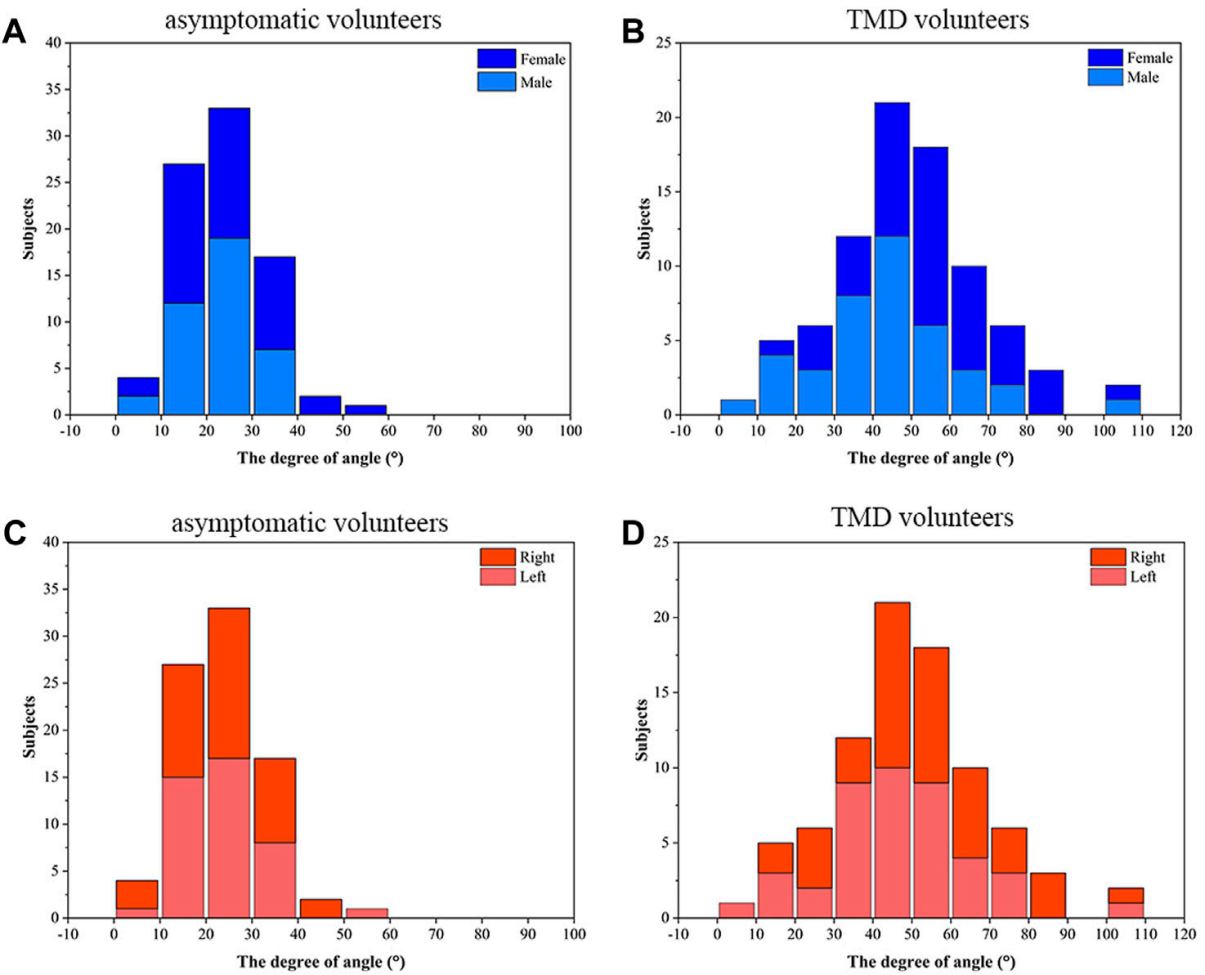
The disc condyle angle measurement results**. (A)** The gender distribution of disc condylar angle in asymptomatic volunteers **(B)** The gender distribution of disc condylar angle in TMD volunteers **(C)** The side distribution of disc condylar angle in asymptomatic volunteers **(D)** The side distribution of disc condylar angle in TMD volunteers.

The disc condyle angle measurement results were displayed in Figure 6. The asymptomatic volunteers have a disc condyle angle of 20°–30° mostly, whereas TMD volunteers have a disc condyle angle of 40°–50°.

**FIGURE 7:**
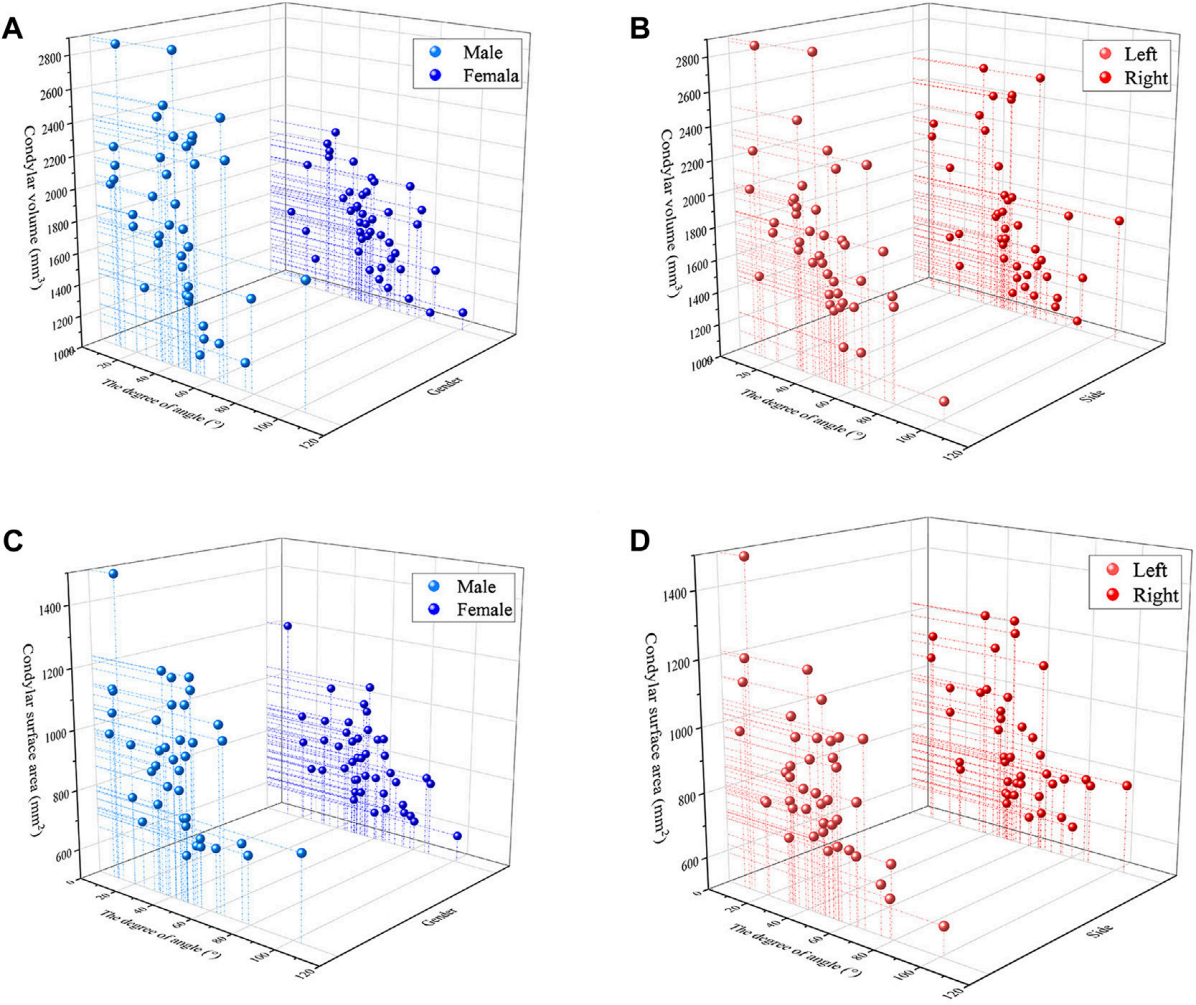
The analysis of correlation **(A)** Condylar volume and the disc condyle angle in males and females **(B)** Condylar volume and the disc condyle angle in left and right **(C)** Condylar surface area and the disc condyle angle in males and females **(D)** Condylar surface area and the disc condyle angle in left and right.

Figure 7 have shown the analysis of correlation between condyle volume surface area and the disc condyle angle in TMD volunteers. There was a significant negative correlation between the volume surface area of the condyle and the disc condyle angle, regardless of gender and side (*p* ˂ 0.05).

## 4 Discussion

TMD is an extremely challenging topic that affects people’s quality of life. Symptoms without imaging findings and imaging findings without symptoms may occur simultaneously in the population. Nevertheless, the necessary imaging examination are needed, such as CBCT and MRI. The advantage of CBCT is to show the condylar bone more clearly (Larheim et al., 2015), while the advantage of MRI is to show the soft tissues such as articular discs, muscles and blood vessels (Xiong et al., 2021). The combination of the advantages of the two examinations can be used in the clinical diagnosis and evaluation of TMD.

In this study, both TMD and asymptomatic volunteers underwent MRI and CBCT. Many studies have shown that the incidence of TMD is significantly correlated with changes in TMJ structure (Ahn et al., 2006; Seo et al., 2020), so joint morphological measurement based on these two examinations is clinically significant and may contribute to TMD diagnosis. Studies have confirmed that the progression of condylar osseous changes is strongly correlated with age (Ogura et al., 2012; de Farias et al., 2015), so the research subjects of this study are limited to young adults to reduce the effect of age on condylar bone.

Incesu proposed a method to determine the position of articular disc by measuring 122 TMJs of 61 TMD patients (Incesu et al., 2004). The anterior displacement of the articular disc was divided into 11°–30° (slight), 30°–50° (mild), 51°–80° (moderate), and over 80° (severe). The articular disc positions of 11°–30° were the most common. In this study, the disc condyle angles were measured by using the condyle apex method, and the distribution frequency of the disc condyle angles was greater in patients with TMD than in asymptomatic volunteers. Asymptomatic volunteers had a disc condyle angle of 20°–30° mostly, whereas TMD volunteers had a disc condyle angle of 40°–50°. This indicates an anterior articular displacement in TMD group compared to asymptomatic volunteers. In addition, here were gender and side differences in the TMD group (*p* < 0.05), but not in the asymptomatic group (*p* > 0.05). This suggests that bilateral articular discs are basically symmetrical in asymptomatic volunteers, as opposed to in the TMD group. In TMD volunteers, the degree of anterior disc displacement was higher in females than in males. This may correspond to the higher clinical incidence of TMD in females (Bagis et al., 2012).

3D visualization of dental imaging seems to be a new direction for future diagnosis. Tecco measured the volume surface area of bilateral condyles in 150 adult Caucasians (Tecco et al., 2010), and found statistically significant differences in condyle volume regardless of gender and side. For the condylar surface area, the difference between the right and the left sides is statistically significant, while those between males and females is not statistically significant. This may be related to racial differences and malocclusion in the study sample. In this study, the volume and surface area of the condyle were measured using CBCT and found to be smaller in TMD volunteers than in asymptomatic group (*p* < 0.05). This suggests that TMD patients may have degenerative remodeling of the condyle, which is consistent with previous findings (Cortés et al., 2011). Gender differences existed in both groups (*p* < 0.05), while side differences only existed in the TMD group (*p* < 0.05). This indicates that the volume surface area of the female condyle is smaller than that of the male, regardless of the presence or absence of TMD. In the TMD group, bilateral condylar morphology may not be consistent due to differences in the degree of disc displacement. Due to different imaging methods, research sample and determination of reference lines, the influence of horizontal condylar axis angle on condylar process morphology is still controversial (Alfaleh, 2021). In this study, the horizontal condylar axial angle was significantly larger in the TMD group (*p* < 0.05). This is consistent with the results of Kurita and Lee’s study (Kurita et al., 2003; Lee et al., 2017). Although there was no gender difference in the asymptomatic group (*p* > 0.05), the horizontal condylar axis angle in females was larger than that in males, this may explain why females are prone to TMD in terms of anatomical structure. Although no significant side differences were found in both two groups (*p* > 0.05), the left horizontal condylar axis angle was larger than the right in TMD group, this may be related to the occurrence of mandibular dyskinesia in some TMD patients.

In terms of correlation, most previous studies have used linear or angular indices to assess the correlation between condylar morphology and articular disc position. Ahn et al. confirmed that patients with disc displacement without reduction has a lower condylar height than those with disc displacement with reduction or normal disc position (Ahn et al., 2006). Guercio et al. had shown that subjects with disc displacement without reduction had a shorter medio-lateral condylar dimension than those with normal disc position. In the anterior-posterior sizes of the condyle, disc displacement without reduction had smaller dimension than disc displacement with reduction (Guercio Monaco et al., 2022). This suggests that condylar morphology is associated with the change of articular disc position. Animal studies corroborated the findings. Li et al. developed a modified model of anterior articular disk displacement in rabbits, where the contour of the condyle in the joint was reshaped and flattened with increasing tensile force and disc displacement (Li et al., 2014).

In this study, statistical analysis revealed a significant negative correlation between condylar volume surface area and oblique sagittal articular disc position in young TMD volunteers (*p* < 0.05), which is consistent with Chang’s research (Chang et al., 2018). The condylar volume of normal disk position, disk displacement with reduction and disk displacement without reduction were compared. The results show that the volume of the condyle decreases as anterior disc displacement increases. One possible explanation for this significant negative correlation is that osteoarthritis is more common in TMD patients with anterior articular disc displacement. As anterior disc displacement advances, bone resorption and remodeling of the condyle occur concurrently, and the trend of bone resorption becomes more obvious (Kurita et al., 2003; Campos et al., 2008).

## 5 Conclusion

The volume surface area of the condyle in TMD volunteers are inversely proportional to the position of the articular disc. This significant negative correlation suggests that the possibility of disc displacement can be considered when poor condylar morphology is found.

## Data Availability

The original contributions presented in the study are included in the article/Supplementary material, further inquiries can be directed to the corresponding authors.
